# Diffusion tensor imaging reveals sex differences in pain sensitivity of rats

**DOI:** 10.3389/fnmol.2023.1073963

**Published:** 2023-03-02

**Authors:** Myeounghoon Cha, Young-Ji Eum, Kyeongmin Kim, Leejeong Kim, Hyeji Bak, Jin-Hun Sohn, Chaejoon Cheong, Bae Hwan Lee

**Affiliations:** ^1^Department of Physiology, Yonsei University College of Medicine, Seoul, Republic of Korea; ^2^Bio-Chemical Analysis Team, Korea Basic Science Institute, Cheongju, Republic of Korea; ^3^Graduate School of Medical Science, Brain Korea 21 Project, Yonsei University College of Medicine, Seoul, Republic of Korea

**Keywords:** diffusion tensor imaging, tractography, sexual dimorphism, pain sensitivity, rat

## Abstract

Studies on differences in brain structure and function according to sex are reported to contribute to differences in behavior and cognition. However, few studies have investigated brain structures or used tractography to investigate gender differences in pain sensitivity. The identification of tracts involved in sex-based structural differences that show pain sensitivity has remained elusive to date. Here, we attempted to demonstrate the sex differences in pain sensitivity and to clarify its relationship with brain structural connectivity. In this study, pain behavior test and brain diffusion tensor imaging (DTI) were performed in male and female rats and tractography was performed on the whole brain using fiber tracking software. We selected eight brain regions related to pain and performed a tractography analysis of these regions. Fractional anisotropy (FA) measurements using automated tractography revealed sex differences in the anterior cingulate cortex (ACC)-, prefrontal cortex (PFC)-, and ventral posterior thalamus-related brain connections. In addition, the results of the correlation analysis of pain sensitivity and DTI tractography showed differences in mean, axial, and radial diffusivities, as well as FA. This study revealed the potential of DTI for exploring circuits involved in pain sensitivity. The behavioral and functional relevance’s of measures derived from DTI tractography is demonstrated by their relationship with pain sensitivity.

## Introduction

It is well known that there are anatomical and functional differences between male and female brains. With the development of technology, non-invasive brain imaging studies on the structural differences between males and females are in progress (Jahanshad and Thompson, 2017; Tyan et al., 2017). However, although functional and structural studies on sexual dimorphism have been conducted, studies on the difference in pain sensation between males and females, especially in the pain signaling pathways, have not been conducted. Furthermore, recent studies have attempted to prove a link between the brain responses to pain and sex differences (Menzler et al., 2011; Koolschijn and Crone, 2013; Cook et al., 2022). However, this is a very challenging question. Because there are large individual differences in brain morphology measurements, differences can be observed even within the same sex; therefore, sex differences are difficult to identify. With the remarkable advances in brain imaging technology, magnetic resonance imaging (MRI) has become a major method to elucidate the structure of the brain and to elucidate the microstructure of the brain (Yang et al., 2021).

Diffusion tensor imaging (DTI), a non-invasive MRI, that measures changes in extra-cellular water molecule diffusion, has been widely applied in neurosurgical operations by providing connectivity information of nerve pathways to help prevent damage to critical nerve pathways during the surgical procedure (Coenen et al., 2015). Quantitative DTI measurements of tractography-derived fiber bundles have been used to detect microstructural defects in several neurological and psychiatric disorders, such as amyotrophic lateral sclerosis, and Alzheimer’s disease (Wang et al., 2006; Fischer et al., 2012; Mesaros et al., 2012). This method is based on the measurement of water diffusion and its directivity, which are influenced by the structure of the surrounding brain tissue. In addition, based on the calculation of the orientation information at each voxel in DTI, tractographic analysis can be used to rebuild the trajectories of the white matter in three-dimensional space (Coenen et al., 2015). Diffusion tensor tractography has orientation-based contrast, allowing for the quantification of integrity and structural connectivity of specific pathways by estimating microstructure or fiber indices along the reconstructed pathways as well as anatomical descriptions of neural pathways (Zhang et al., 2020).

The sexual dimorphism observed in brain anatomy has been analyzed in several studies focusing on the ratio between the gray and white matters, regional brain volume, and overall size (Nopoulos et al., 2000; Giedd et al., 2012; Koolschijn and Crone, 2013; Cook et al., 2022). It should be noted that while differences in brain size are usually due to sex differences in body weight, these absolute differences in brain size remain even after adjusting for differences in body size. Sexual dimorphism is reflected in functional connectivity of the brain. First, sex differences in connectivity coincide with areas representing volumetric differences based on sex, including the amygdala, frontal, and temporal lobes (Wu et al., 2013; Barendse et al., 2018). In addition, differences in network level have been reported in resting-state regional connections (Allen et al., 2011). Finally, males have stronger inter-network connections but weaker inter-hemispheric connections (Allen et al., 2011; Satterthwaite et al., 2015). In general, evidence indicative of sex differences in overall brain size, gray and white matter ratios, and regional brain volumes suggests the need to investigate sexual dimorphism in functional connectivity in pain-sensitive brain regions. Although it is a very interesting area of research, studies of sex differences in the brain that are sensitive to pain have not yet been conducted.

This study aimed to identify sex differences of structural connectivity by using DTI data from the brains of adult rats. We attempted to isolate the most stable and salient features that could predict sex differences, along with differences in brain connectivity between males and females. Based on the results, it is possible to further characterize brain functions and connectivity patterns based on sex differences. At first, we hypothesized that differences demonstrated using DTI analysis would be strong enough to classify brains based on sex. In addition, we also hypothesized that the most notable differences in connectivity would exist in differences in connectivity between brain regions responsible for pain information processing.

## Materials and methods

### Animals

All animal experiments were conducted in accordance with the National Institutes of Health guidelines. The experimental procedures were reviewed and approved by the Institutional Animal Care Use Committee (IACUC) of the Yonsei University Health System (permit no. 2019-0225). Adult male and female rats (240 ± 10 g, 7–8 weeks old; Sprague Dawley rats, Harlan, Koatec, Pyeongtaek, Korea) allocated for the experiments were individually housed and maintained on a 12/12 h light-dark cycle at 22 ± 2°C and 50–60% humidity. Food and water were available *ad libitum*.

### Mechanical threshold measurement

Behavioral tests were conducted to compare the mechanical thresholds (MT) between male and female rats (male, *n* = 8; female, *n* = 8). MT was measured three times over 3 days using an electronic von Frey (no. 38450; Ugo Basile, Varese, Italy). The rats were individually placed in acrylic cages on a wire mesh and allowed to habituate for 15 min. The test was repeated seven times per rat. The average values of the data were obtained, except for the minimum and maximum values. The behavioral testing of male and female rats was performed at different times and the acrylic cage was wiped with 70% alcohol for each measurement before use, for each test. All behavioral tests were performed by a researcher who was blinded to the experimental groups.

### Fixation and mounting procedure

For DTI, rats were euthanized using urethane and perfused with phosphate buffered saline (PBS, pH 7.4) followed by 4% paraformaldehyde (PFA) in PBS. The brains were subsequently extracted from the skull and post-fixed (for 24 h) in 4% PFA. They were then rinsed in PBS and stored at 4°C in fluorinert (FC-770, Sigma, St. Louis, MI, US) until being used for DTI. Before MRI, the brains were embedded in 10 mL syringes (Fisher Scientific, Hampton, NH, US), with an approximate outer diameter of 1.6 cm and a length of 2 cm from cap to tip. Each brain was immersed in liquid fluoride and fixed without shaking, using a syringe, and the tip of the syringe was fixed with silicone.

### Data acquisition

All imaging was performed on a 9.4 T horizontal Biospec bore scanner (BioSpec 94/20; Bore diameter: 20 cm, Bruker, BioSpin, Ettlingen, Germany). A circularly polarized transmit/receive 1H volume coil was used to obtain maximum resolution for the DTI/tractography experiments. The data were collected in the axial orientation, with the read-out direction oriented to the long axis of the tube. For each tube of the brain, T2-weighted images were acquired at 100 μm isotropic resolution (TE = 26 ms, matrix 256 × 256). Diffusion images were acquired on a Bruker BioSpin MRI GmbH scanner using the DtiEpi SpinEcho sequence (TE = 32 ms, and TR = 12500.001 ms). The diffusion encoding duration was 4 ms. A DTI diffusion scheme was used, and a total of 30 diffusion sampling directions were acquired. The *b*-value was 3,000.0 s/mm^2^, in-plane resolution 0.134375 mm, and slice thickness 0.5 mm. The *b*-table was checked using an automatic quality control routine to ensure accuracy, and the diffusion tensor was calculated (Schilling et al., 2019).

### Region of interest (ROI) selection and image processing

Eight brain regions (ACC, anterior cingulate cortex; PFC, prefrontal cortex; IC, insular cortex; S1, primary somatosensory cortex; S2, secondary somatosensory cortex; VP, ventral posterior thalamic nucleus; PAG, periaqueductal gray matter; Amy, amygdala) associated with pain information processing were selected as ROIs for tractography (Baliki et al., 2006; Tracey and Mantyh, 2007; De Ridder et al., 2021). High-resolution SIGMA rat brain template (Barrière et al., 2019) and Paxinos and Watson atlas were used (Paxinos and Watson, 2005). The ROI masks were acquired from the SIGMA atlas using Atlas Normalization Toolbox with Elastix 2 (ANTx2, University Medicine Berlin, Berlin, Germany). DTI data were processed using ANTx2, the Functional Magnetic Resonance Imaging of the Brain (FMRIB) software library version 6.0.2 (FSL, created by the Analysis Group, Oxford, UK), and MRtrix3 (^1^ Tournier et al., 2019). Using ANTx2, format conversion to Neuroimaging Informatics Technology Initiative, re-orientation to SIGMA space (Barrière et al., 2019), and extraction of B0 images were performed. Subsequently, all data were linearly registered and spatially normalized into SIGMA space using FMRIB’s Linear Image Registration Tool function (Barrière et al., 2019). DTI data were denoised using MRtrix3 and corrected for distortions and motion artifacts using the eddy-correct tool in FSL (Im et al., 2021).

### Tractography

Tractography analysis was performed using DSI Studio.^2^ Deterministic tractography was then performed using the following global parameters: angular threshold = 60°, step size = 0.05 mm, minimum length = 1 mm, terminate if = 600,000 seeds). The tracking threshold was calculated using DSI Studio to maximize the variance between the background and foreground. The maximum length was defined differently, considering the anatomical distance between two ROIs. ROI-based tracking was used to investigate the connectivity of the brain regions associated with pain information processing. The tracking resulted in the number of streamlines seeded on one ROI targeting the other ROI in the ipsilateral hemisphere. Corresponding values for the DTI indices [fractional anisotropy (FA); mean diffusivity (MD); axial diffusivity (AD); and radial diffusivity (RD)] were extracted from the voxels included in the tracked streamlines.

### Statistical analysis

All statistical analyses were performed using SPSS version 28 (IBM Corp., Armonk, NY, USA). The average value of the mechanical threshold measured for 3 days was used to assess pain sensitivity. Prior to analysis, we tested whether data violated the assumption of normality using Shapiro-Wilk test. The result revealed that there were no significant variables, and our data follow normal distribution. Sex differences in pain sensitivity and DTI indices were analyzed using Mann-Whitney *U* test. *P*-values were adjusted for multiple comparisons using the Benjamini–Hochberg procedure; false discovery rate (FDR) less than 0.05 was considered as statistically significant. We also investigated whether there was an association between DTI indices in each streamline and pain sensitivity using Pearson’s correlation analysis. All the tests were two-tailed, and the threshold for statistical significance was set at *p* < 0.05.

## Results

### Mechanical threshold

**FIGURE 1:**
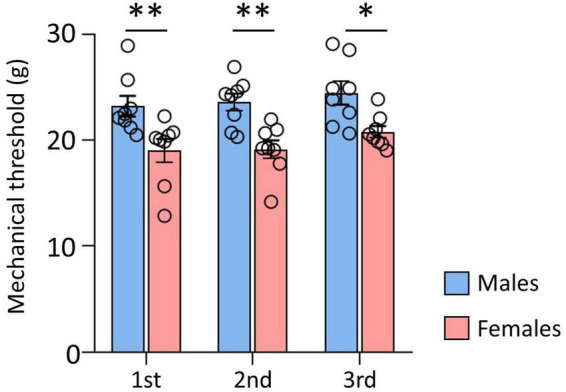
Comparison of mechanical thresholds (MT) between male and female. There were significant differences between male and female rats. Male rats showed significantly higher MT than female rats. Data are presented as means ± standard error of the mean. ^**^p < 0.001 and *p < 0.05 vs. female rats determined using Student’s *t*-test.

The data of the mechanical thresholds measured before DTI, to compare the sex differences, are shown in Figure 1. The results of three repeated behavioral tests indicated that the threshold of male rats was significantly higher than that of female rats (male: 23.05 ± 1.23; female: 19.74 ± 0.67, *p* < 0.001). These data indicated a withdrawal threshold difference between male and female rats.

### Brain fiber tracts and ROI

**FIGURE 2:**
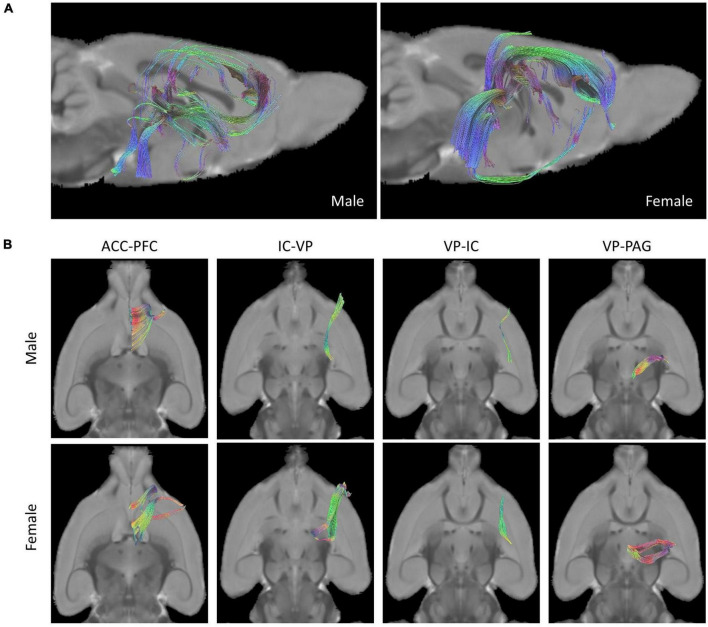
Tractographies of male and female rat brains. **(A)** Representative placement of tracks drawn in pairs of ROIs of “seed” and “end” and composition of fibers done with tractography (Left: Male, Right: Female). **(B)** Female rats showed more tracts in the tractography analysis of ROIs than male rats in the analysis of four seed-end tractographies (Upper: Male, Lower: Female).

A comparison of rat brain tracts in males and females was analyzed by determining the seed-end regions in eight ROIs. Representative male and female rat brain tractography are shown in Figure 2. In Figure 2A, we compared how different each tract appeared in male and female. In addition, the differences between the four tracks showing statistical significance were compared in Figure 2B.

### Fractional anisotropy, mean, axial, and radial diffusivities

**FIGURE 3:**
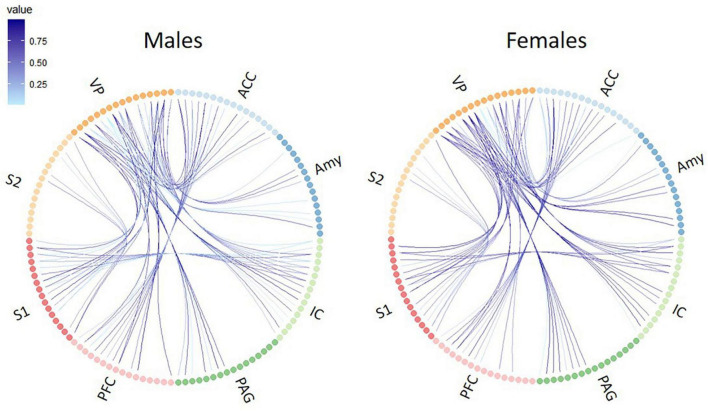
Circular connectome graphs representing the fractional anisotropy (FA) values and connections between pain-related brain regions in male **(left)** and female **(right)** rats. Each line represents connectivity between a pair of brain regions and the intensity of the lines indicate the FA values. ACC, anterior cingulate cortex; PFC, prefrontal cortex; IC, insular cortex; S1, primary somatosensory cortex; S2, secondary somatosensory cortex; VP, ventral posterior thalamic nucleus; PAG, periaqueductal gray matter; Amy, amygdala.

Using whole-brain DTI, we discovered significant regional microstructural sex differences in the ACC-PFC, VP-IC, VP-PAG, and IC-VP trajectories (Mann-Whitney *U* test between male and female rats; ACC-PFC: U = 4, *p* = 0.003; VP-IC: *U* = 11, *p* = 0.049; VP-PAG: *U* = 11, *p* = 0.049; IC-VP: *U* = 3, *p* = 0.010): females showed significantly higher FA values than male rats in these regions (*p* < 0.05) (Table 1). In addition, the FA values of the ACC-PFC of female rats showed the highest difference from that of males. Each significant value is indicated in bold font in Table 1. Figure 3 illustrates regional connections in males and female rats among eight different regions (ACC, IC, PFC, S1, S2, VP, Amy, and PAG), using circular connectome. Interconnectivity is expressed as lines and is shown in colors varying in intensity according to the FA values. These results demonstrate sex differences in connectivity of these brain regions. Data obtained from trajectories using DTI showed that the success rate of fiber path generation varied. The highest success rate was 100% and the lowest was 25%; however, there was no correlation between the success rates and measures obtained through tractography. The success rates of the tractography connections are summarized in Table 2.

**TABLE 1:** Mean value and standard error of the mean of fractional anisotropy, mean, axial, and radial diffusivities between male and female rats.

			FA			MD			AD			RD		
**Pathway**	**Gender**	**N**	**Mean**	**SEM**	**U**	**Mean**	**SEM**	**U**	**Mean**	**SEM**	**U**	**Mean**	**SEM**	**U**
**ACC-PFC**	**Male**	**8**	0.256	0.008	4^**^	0.316	0.009	31	0.401	0.013	20	0.274	0.008	23
	**Female**	**8**	0.321	0.015		0.322	0.006		0.437	0.012		0.264	0.005	
IC-S1	Male	7	0.239	0.016	5	0.32	0.01	7	0.402	0.011	5	0.28	0.01	13
	Female	4	0.278	0.013		0.341	0.008		0.445	0.014		0.289	0.006	
S1-S2	Male	5	0.251	0.023	12	0.316	0.009	10	0.398	0.017	12	0.275	0.01	12
	Female	5	0.251	0.025		0.316	0.011		0.395	0.014		0.277	0.012	
VP-ACC	Male	6	0.285	0.015	13	0.302	0.011	13	0.394	0.014	14	0.256	0.011	13
	Female	5	0.288	0.016		0.31	0.009		0.406	0.015		0.262	0.008	
VP-Amy	Male	4	0.31	0.032	5	0.308	0.008	6	0.411	0.019	7	0.256	0.004	4
	Female	4	0.347	0.01		0.307	0.014		0.425	0.017		0.248	0.013	
**VP-IC**	**Male**	**8**	0.253	0.017	11*	0.31	0.01	25	0.391	0.012	21	0.27	0.01	20
	**Female**	**7**	0.301	0.011		0.306	0.011		0.406	0.012		0.256	0.011	
**VP-PAG**	**Male**	**7**	**0.229**	0.014	11*	0.296	0.004	24	0.365	0.009	20	0.262	0.003	18
	**Female**	**8**	**0.277**	0.015		0.297	0.008		0.381	0.012		0.254	0.007	
VP-PFC	Male	3	0.229	0.025	1	0.326	0.008	0	0.404	0.016	2	0.287	0.005	0
	Female	2	0.284	0.03		0.304	0.002		0.395	0.01		0.258	0.008	
VP-S1	Male	6	0.277	0.025	15	0.306	0.012	17	0.395	0.013	16	0.262	0.013	18
	Female	6	0.297	0.031		0.311	0.013		0.406	0.013		0.263	0.015	
ACC-VP	Male	5	0.272	0.033	9	0.309	0.008	11	0.398	0.015	10	0.264	0.01	12
	Female	5	0.287	0.009		0.316	0.011		0.411	0.019		0.268	0.008	
Amy-VP	Male	4	0.274	0.025	5	0.31	0.009	4	0.399	0.011	5	0.265	0.01	6
	Female	4	0.293	0.022		0.326	0.012		0.428	0.024		0.275	0.007	
**IC-VP**	**Male**	**7**	0.222	0.009	3*	0.309	0.008	18	0.379	0.012	14	0.274	0.007	14
	**Female**	**6**	0.268	0.01		0.316	0.01		0.407	0.017		0.271	0.007	
PFC-VP	Male	3	0.229	0.006	2	0.322	0.01	1	0.398	0.013	1	0.284	0.008	2
	Female	2	0.218	0.02		0.304	0.004		0.371	0.012		0.27	0.001	
S1-VP	Male	4	0.278	0.03	7	0.316	0.008	4	0.412	0.02	7	0.268	0.006	5
	Female	4	0.299	0.006		0.309	0.02		0.407	0.029		0.26	0.016	

ACC, anterior cingulate cortex; PFC, prefrontal cortex; IC, insular cortex; S1, primary somatosensory cortex; S2, secondary somatosensory cortex; VP, ventral posterior thalamic nucleus; PAG, periaqueductal gray matter; Amy, amygdala; FA, fractional anisotropy; MD, mean diffusivity; AD, axial diffusivity; RD, radial diffusivity. Data are presented as mean ± s.e.m. *p < 0.05 and **p < 0.01 (*U*; PDR-corrected Mann-Whitney *U*-test). Statistically significant values are indicated in bold.

**TABLE 2:** Success rate of tractography for each brain connection.

Brain tracts	Successs rate based on tractography	Success rate (%)
ACC-PFC	16/16	100%
IC-S1	11/16	68.75%
S1-S2	13/16	81.25%
VP-ACC	10/16	62.50%
VP-Amy	11/16	68.75%
VP-IC	9/16	56.25%
VP-PAG	15/16	93.75%
VP-PFC	15/16	93.75%
VP-S1	5/16	31.25%
ACC-VP	12/16	75%
Amy-VP	11/16	68.75%
IC-VP	8/16	50%
PFC-VP	4/16	25%
S1-VP	8/16	50%

### Relation between DTI and mechanical threshold

**FIGURE 4:**
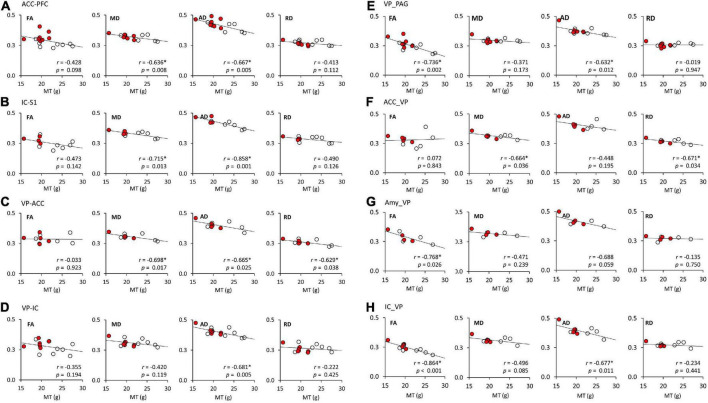
The linear regression analysis of DTI tractography data (FA, MD, AD, and RD) and mechanical thresholds (MT). **(A–H)** Represent the FA, MD, AD, and RD values of each seed-end region. The red or white dots in the graphs represent data from female or male rats, respectively. Pearson’s correlation coefficients (*r*) and *p* values are provided on the bottom right side of each graph. *p < 0.05 indicates a significant correlation. ACC, anterior cingulate cortex; PFC, prefrontal cortex; IC, insular cortex; S1, primary somatosensory cortex; S2, secondary somatosensory cortex; VP, ventral posterior thalamic nucleus; PAG, periaqueductal gray matter; Amy, amygdala; FA, fractional anisotropy; MD, mean diffusivity; AD, axial diffusivity; RD, radial diffusivity.

A linear regression model was used to investigate the relationship of FA, MD, AD, and RD of each brain tract and MT (Figure 4 and Table 3). Figure 4 shows the significant associations that were observed between MT and FA, MD, AD, and RD in pain pathway tracts. In the ACC-PFC, MD and AD were significantly correlated with MT (MD: *r* = −0.636, *p* = 0.008; AD: *r* = −0.667, *p* = 0.005). MD and AD in the IC-S1 also showed significant correlations with MT (MD: *r* = −0.715, *p* = 0.013; AD: *r* = −0.858, *p* = 0.001). In the VP-ACC, MD, AD, and RD were significantly correlated with MT (MD: *r* = −0.698, *p* = 0.017; AD: *r* = −0.655, *p* = 0.025; RD: *r* = −0.629, *p* = 0.038). In the VP-IC, only AD showed a significant correlation with MT (AD: *r* = −0.681, *p* = 0.005).

**TABLE 3:** Pearson’s correlation coefficients (*r*) between mechanical threshold and DTI indices.

	FA	MD	AD	RD
ACC-PFC	-0.428	**−0**.636**	**−0**.667**	-0.413
IC-S1	-0.473	**−0**.715*	**−0**.858**	-0.490
S1-S2	0.166	-0.427	-0.197	-0.482
VP-Amy	-0.602	-0.385	-0.586	-0.114
VP-IC	-0.355	-0.420	**−0**.681**	-0.222
VP-PAG	**−0**.736**	-0.371	**−0**.632**	-0.019
VP-PFC	-0.658	0.651	0.077	0.794
VP-S1	-0.005	-0.336	-0.375	-0.257
ACC-VP	0.072	**−0**.664*	-0.448	**−0**.671*
Amy-VP	**−0**.768*	-0.471	-0.688	-0.135
IC-VP	**−0**.864***	-0.496	**−0**.677*	-0.234
PFC-VP	0.401	0.179	0.243	0.110
S1-VP	-0.071	-0.465	-0.407	-0.473

*p < 0.05, **p < 0.01, and ***p < 0.001. Statistically significant values are indicated in bold.

FA and AD in the VP-PAG were significantly correlated with MT (FA: *r* = −0.736, *p* = 0.002; AD: *r* = −0.632, *p* = 0.012). MD and RD in the ACC-VP also showed significant correlations with MT (MD: *r* = −0.664, *p* = 0.036; RD: *r* = −0.671, *p* = 0.034). In the Amy-VP, only FA showed a significant correlation with MT (FA: *r* = −0.768, *p* = 0.026). Finally, in the IC-VP, FA and AD showed a significant correlation with MT (FA: *r* = −0.864, *p* < 0.001; AD: *r* = −0.677, *p* = 0.011). A few data points did not show any significant relationship between DTI and MT; these are summarized in Supplementary Figure 1.

## Discussion

In this study, we used DTI tractography approach to determine whether differences in MT in male and female rats were related to differences in pain pathways in the rat brain. Our results demonstrated the utility of quantitative tractography in analyzing sexual dimorphism in pain pathways. This application demonstrates that differences in connectivity between individual brain regions are related to differences in the degree of pain perception between male and female rats.

### Pain perception and neurological pathways in the brain

Recently, increasing evidence indicates the sex differences in pain sensitivity (Racine et al., 2012; Vasung et al., 2020; Dawes and Bennett, 2021). The difference in pain perception according to sex could be decisive consideration for pain control in clinical pain treatment. Our pain behavior results show that there are differences in pain perception between males and females in normal animals, and involve specific differences, including genetic differences that exist between the sexes. Previous studies have focused on biological mechanisms, including hormonal influence (Craft, 2007; Cairns and Gazerani, 2009), and tried to explain the causes of differences in pain sensitivity between sexes by psychosocial mechanisms such as social support, positive self-statement, emotion–focused therapy, and cognitive reinterpretation (Robinson et al., 2001; Racine et al., 2012). However, even this evidence is insufficient to clearly explain sex differences in pain.

Since the first imaging studies of pain began in the 1970s (Lassen et al., 1978), advances in technology have provided diverse evidence of the interaction between neuronal evidence of brain activity and pain response. Previous positron emission tomography (PET) and functional MRI (fMRI) studies have examined the neural processing of pain evoked by stimuli on the skin, confirming that multiple brain regions are activated. In this study, we analyzed the sex differences in connectivity between eight pain-related brain regions (ACC, PFC, IC, S1, S2, VP, Amy, and PAG) in limbic and subcortical areas using DTI. In particular, higher FA value of the ACC-PFC in female rats suggests that emotional distress may have a significant impact on females. Furthermore, higher FA value of the VP-IC, IC-VP, and VP-PAG in female rats as compared to male rats can be considered to mean that the VP plays a important role in nociception in females. Numerous studies on pain processing have recognized the activation of S1 and S2 regions and this evidence has led to the understanding that during the processing of nociceptive stimuli, S1 and S2 regions of the brain perceive the sensory features of pain (Coghill et al., 1999; Craig, 2002). Additionally, the ACC and IC, components of the limbic system, found to be activated in most PET or fMRI studies of thermal and mechanical pain, are implicated in the emotional processing of pain (Orenius et al., 2017; Cha et al., 2020; Henderson et al., 2020; Shi et al., 2021). It has been reported that the parietal association area and the prefrontal cortex are involved in the processing of thermal pain and are related to cognitive factors such as memory or stimulus evaluation (Strigo et al., 2003). Subcortical activation has also been reported, most notably in the ventroposterolateral (VPL) and ventroposteromedial (VPM) nuclei in the thalamus (Sanganahalli et al., 2022), basal ganglia (Raver et al., 2020), and cerebellum (Bermo et al., 2020). It has been known that the amygdala is also central to the emotional processing of sensory stimuli, including pain (Gandhi et al., 2020). Recent findings indicate that individual variations in emotional processes are closely related to pain, and studies on functional connectivity for individual patterns that make individuals sensitive to emotionally controlled pain facilitation are ongoing (Fillingim, 2017; McIlwrath et al., 2020). Studies in humans have indicated that the location and intensity of nociceptive input are encoded in brain regions including the posterior insular cortex and S1, S2/operculum (Woo et al., 2017). On the contrary, the affective aspect of pain integrates neural inputs from limbic structures including the amygdala and sensory brain regions, and is associated with brain regions such as the occipital and parietal cortex, anterior cingulate cortex, and frontal insula (Tracey and Mantyh, 2007). In this study, our DTI results showed clear sex differences by analyzing the connectivity of eight areas related to pain, and these differences in brain connectivity could provide clues as to whether pain varies depending on sex.

### Quantitative tractography and pain behavior

Most FA and DTI studies on pain have focused on examining the differences in microstructure of white matter in pain versus non-pain models and investigating the correlation between FA and pain levels (Martucci et al., 2014; Knudsen et al., 2018). In this study, we attempted to find a correlation between the differences in perceived pain between males and females and the tractography of DTI. Structurally, female fetuses show smaller inferior frontal gyrus and cingulate volumes (Vasung et al., 2020). As females grow, they exhibit strengthening of long-distance connections, particularly between the prefrontal networks and the subcortical, visual, and cerebellar networks. Alternatively, males exhibit enhanced connections within localized areas, including the frontal lobes and cerebellum (Cook et al., 2022). Studies of the responses of medial and lateral thalamic neurons to noxious and harmless stimuli have revealed that the medial and lateral pathways specifically process the sensory and emotional aspects of nociception (Groh et al., 2017, 2018). Indeed, most neurons in VP respond to mechanical or thermal stimuli in a differential manner, with a low firing rate at harmless stimulus and a high firing rate at noxious stimulus (Masri et al., 2009; Demori et al., 2022). Our DTI results show the difference in brain development and brain connectivity between males and females, providing a clue to explain the reason for the difference in sensitivity to pain between sexes. As shown in Table 3 and Figure 4, significant correlations were found between the pain sensitivity of rats and the measures obtained through tractography analysis. Our results indicated that increased pain sensitivity in males and females was associated with increased FA values of IC-VP, VP-PAG, and Amy-VP. These findings suggest that the effect of sex difference might be involved in brain connectivity.

The results of our study indicated that sex differences in brain connectivity associated with pain sensitivity can be detected using DTI tractography, and there were significant sex differences in the association between the two measures as demonstrated through linear analysis. The most influential and consistent differences were observed in the brain regions with known sex differences in male and female rats, particularly in the ACC-PFC connections, and the largest differences were found between the VP and other brain regions. These results suggest that different networks may be involved in pain information processing based on gender, giving rise to sex differences in the perception of pain. The observed differences have important implications for understanding sex differences in pain sensitivity and may help understand clinical approaches to pain and structural differences between male and female brains. In addition, more pronounced differences may be observed when statistical analysis would be performed on a larger sample to investigate the relationship between sex, pain sensitivity, and DTI measures.

## Data availability statement

The original contributions presented in this study are included in the article/Supplementary material, further inquiries can be directed to the corresponding authors.

## Ethics statement

This study was performed in line with the principles of the Declaration of Helsinki. The experimental procedures were reviewed and approved by the Institutional Animal Care Use Committee (IACUC) of the Yonsei University Health System (permit no. 2019-0225).

## Author contributions

LK, HB, and KK prepared the materials. MC, Y-JE, and KK performed the data collection and analysis. J-HS, CC, and BL performed the supervision. MC wrote the first draft of the manuscript. All authors contributed to the study conception, design, commented on subsequent versions of the manuscript, read, and approved the final manuscript.

## Footnotes

1
https://www.mrtrix.org

2
https://dsi-studio.labsolver.org
