# Supplementary material for: Diffusion tensor imaging reveals sex differences in pain sensitivity of rats
Source: Front Mol Neurosci. 2023 Mar 2;16:1073963. doi: 10.3389/fnmol.2023.1073963 (PMC10017469; doi:10.3389/fnmol.2023.1073963)
Supplement: Supplementary file 1 [file Data_Sheet_1.docx]

Supplementary Figures


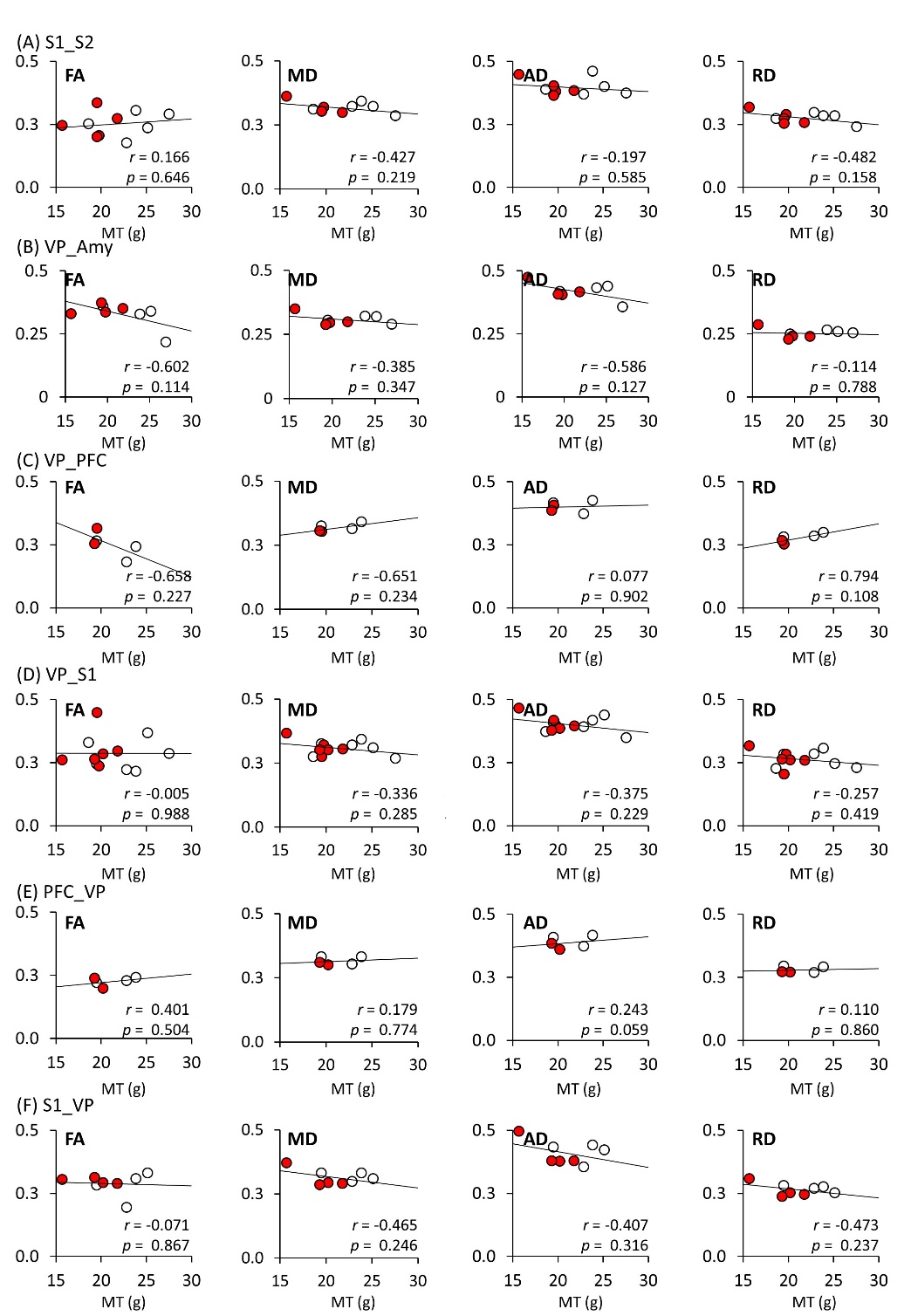


**Supplementary Figure 1.** Non-significant values of linear regression analysis of measures obtained through DTI tractography (FA, MD, AD, and RD) and mechanical thresholds (MT). The red or white dots in each graph represent data from female or male rats, respectively. Pearson’s correlation coefficients (r) and p values are provided on the bottom right side of each graph.
